# Role of complement and antibodies in controlling infection with pathogenic simian immunodeficiency virus (SIV) in macaques vaccinated with replication-deficient viral vectors

**DOI:** 10.1186/1742-4690-6-60

**Published:** 2009-06-21

**Authors:** Barbara Falkensammer, Barbara Rubner, Alexander Hiltgartner, Doris Wilflingseder, Christiane Stahl Hennig, Seraphin Kuate, Klaus Überla, Stephen Norley, Alexander Strasak, Paul Racz, Heribert Stoiber

**Affiliations:** 1 Department of Hygiene, Microbiology and Social Medicine, Innsbruck Medical University, Fritz-Pregl-Str. 3, 6020 Innsbruck, Austria; 2 Department of Infection Models, German Primate Centre, Kellnerweg 4, 37077 Göttingen, Germany; 3 Department of Molecular and Medical Virology, Ruhr-University, Bochum, Universitätsstraße 150, 44801 Bochum, Germany; 4 Robert Koch-Institut, Nordufer 20, 13353 Berlin, Germany; 5 Department for Medical Statistics, Informatics and Health Economics, Innsbruck Medical University, Schöpfstr. 41/1, 6020 Innsbruck, Austria; 6 Department of Pathology and Körber Laboratory for AIDS Research, Bernhard-Nocht-Institute for Tropical Medicine, Postfach 30 41 20, 20324 Hamburg, Germany

## Abstract

**Background:**

We investigated the interplay between complement and antibodies upon priming with single-cycle replicating viral vectors (SCIV) encoding SIV antigens combined with Adeno5-SIV or SCIV pseudotyped with murine leukemia virus envelope boosting strategies. The vaccine was applied via spray-immunization to the tonsils of rhesus macaques and compared with systemic regimens.

**Results:**

Independent of the application regimen or route, viral loads were significantly reduced after challenge with SIVmac239 (p < 0.03) compared to controls. Considerable amounts of neutralizing antibodies were induced in systemic immunized monkeys. Most of the sera harvested during peak viremia exhibited a trend with an inverse correlation between complement C3-deposition on viral particles and plasma viral load within the different vaccination groups. In contrast, the amount of the observed complement-mediated lysis did not correlate with the reduction of SIV titres.

**Conclusion:**

The heterologous prime-boost strategy with replication-deficient viral vectors administered exclusively via the tonsils did not induce any neutralizing antibodies before challenge. However, after challenge, comparable SIV-specific humoral immune responses were observed in all vaccinated animals. Immunization with single cycle immunodeficiency viruses mounts humoral immune responses comparable to live-attenuated immunodeficiency virus vaccines.

## Background

Beside cellular immune responses, humoral immunity is considered a key component in AIDS vaccine development. Already during early stages of viral infection, anti-envelope (env) antibodies (Abs) are thought to reduce viremia [1-3]. Their effector functions are still not completely defined. Some of such neutralizing antibodies (nAbs) may inhibit viral entry either by interfering with structures of the gp120/gp41 complex [4] or with env-epitopes that bind to chemokine receptors. Alternatively, they may cross-link virus particles and induce clearance of immune-complexed viruses by phagocytosis. Additionally, antibody dependent cellular cytotoxicity (ADCC) is thought to appear early during acute infection [5] and can also be detected at later stages of disease progression. ADCC has been studied in the SIV monkey model, was associated with the control of HIV in infected humans [6-8] and may contribute to a slower disease progression in long-term non-progressors [9].

A further arm of the humoral immune response is the complement system as an important mechanism of innate immune defence. Complement (C) has been shown to enhance the activity of nAbs [10]. In synergy to the binding of Abs to viruses, C3 deposition, opsonization and immune complex formation are suggested to contribute to reduced viral infection rates. There is evidence that C-mediated lysis contributes mainly at early stages of HIV-1 infection to viremia control [11-13].

A major focus of current research is the design of safe and efficient vaccines providing a high level of protection against HIV. A promising approach is the application of replication-deficient single-cycle immunodeficiency viruses (SCIV) [14,15]. Upon application, these viral constructs undergo only one single round of replication resulting in the production of non-infectious virus-like particles *in vivo*. The induced immune response is thought to protect from challenge by clearing infected cells.

A non-invasive application of live-attenuated SIV vaccines to the mucosa via the tonsils has been established. This approach induced protection against challenge with homologous SIV and SHIV, a SIV/HIV-1 hybridvirus containing HIV-1 envelope in the SIV backbone [16,17]. Although effective, the delivery of attenuated retroviruses is not feasible in humans due to safety concerns [18,19]. Thus, we adopted a heterologous prime-boost regimen through priming with SCIV and boosting with Adeno5 (Ad5)-SIV or SCIV. The vectors were either given systemically or exclusively mucosally.

To elucidate the induction of immune responses upon vaccination, 12 rhesus macaques were primed with SCIV. Four of the animals received the immunizations via the tonsillar route and eight intravenously (iv) (Table 1). The SCIVs used for priming were pseudotyped with the G protein of vesicular stomatitis virus (VSV-G) to favour and enhance expression of SIV-virus like particles in a broad spectrum of cells, including dendritic cells [20]. The four tonsillar and four of the iv immunized monkeys were boosted with two adenoviral vectors expressing SIV-gag-pol, and SIV env and rev, respectively. The remaining four iv SCIV immunized animals were boosted with SCIV pseudotyped with amphotropic murine leukemia virus envelope (SCIV [MLV]), since we previously observed rapid induction of VSV-G-nAbs after immunization with VSV-G pseudotyped SCIVs [15].

**Table 1:** Immunization regimen

		weeks post immunization			
	monkeys	0	4	8	12

group 1	12127	SCIV [VSV-G]	SCIV [VSV-G]	Ad5-SIV	Ad5-SIV
	12128	tonsillar	tonsillar	tonsillar	tonsillar
	12131	1.8 × 10^9, a^	1.2 × 10^8, a^	1 × 10^11, b^	1 × 10^11, b^
	12137				

group 2	12133	SCIV [VSV-G]		Ad5-SIV	
	12136	intravenous		intramuscular	
	12142	2 × 10^9, a^		6 × 10^11, b^	
	12143				

group 3	12132	SCIV [VSV-G]		SCIV [MLV]	
	12138	intravenous		intravenous	
	12139	2 × 10^9, a^		3 × 10^7, a^	
	12140				

group 4a	12129			Ad5GFP tonsillar	Ad5GFP tonsillar
	12130			1 × 10^11, c^	2 × 10^11, c^

group 4b	12134			Ad5GFP intramuscular	
	12141			6 × 10^11, c^	

^a^infectious units/ml

^b^number of particles per construct

^c^number of particles

The results of the systemic spread of SCIV after oral immunization, as well as analyses concerning the cellular immune responses, immunohistochemical and *in situ *hybridisation assays have been recently published by Stahl-Hennig et al. [21]. In the present study, we characterized the humoral immune response in immunized and challenged rhesus macaques and investigated the contribution of the induced neutralizing and non-neutralizing antibodies, C-deposition on the viral surface and C-mediated lysis with regard to the control of retroviral infection.

## Results

### Viral load levels

**Figure 1:**
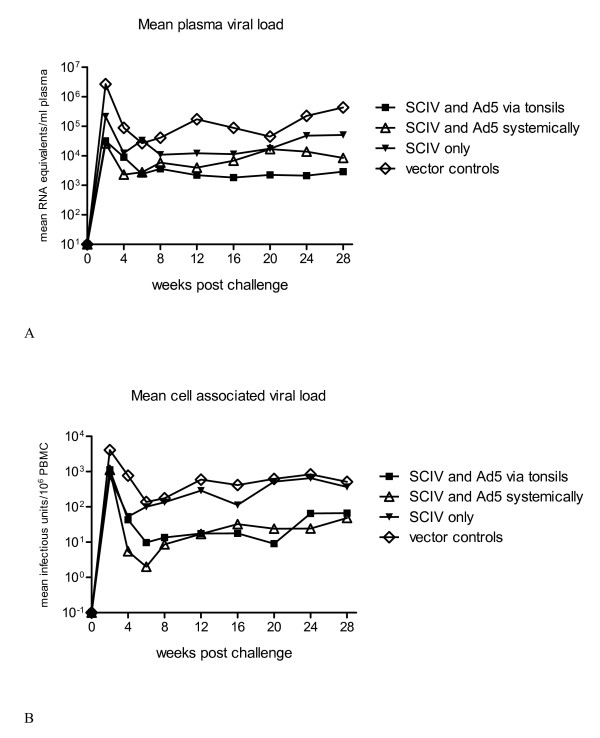
**Determination of plasma and cell-associated viral loads**. The mean plasma viral load levels (A) and mean cell-associated viremia (B) of three immunized and one control cohort are shown after tonsillar challenge with pathogenic SIVmac239. Viral RNA was determined by real-time PCR whereas cell-associated viremia was analysed by a limiting dilution co-culture assay with mononuclear cells from blood.

At 20 weeks post infection (wpi) all vaccinated monkeys and the respective control animals were challenged with pathogenic SIVmac239 via the tonsils. Viremia peaked approximately 2 weeks post challenge (wpc) as determined in plasma and by analyzing cell-associated SIV (Figure 1A, B). Peak RNA levels of SIV in immunized monkeys were significantly reduced by 1 to 2 log compared to control monkeys (p < 0.03 for all comparisons, Figure 1A). The difference among the vaccinated animals in cell-associated viral loads was less pronounced and statistically not significant 2 wpc (p = 0.09, Figure 1B). Plasma and cell-associated viral loads correlated over the complete observation period. During the chronic phase of infection (16 wpc, 28 wpc) monkeys of group 1 and 2 could significantly reduce plasma viremia compared to the control group (all p < 0.05). After 2 wpc differences between the control cohort and group 3 as well as differences between the three vaccinated cohorts were statistically not significant.

### SIV neutralizing antibodies

By a yield reduction assay using SIVmac251, the first detectable nAbs were measurable in group 2 and 3 with mean fold inhibitions of 171.8 and 110.5, respectively, 4 weeks after the first boost (12 wpi). In group 1, nAbs remained undetectable upon immunization. However, after challenge with pathogenic SIVmac239, nAbs rapidly increased, and by 8 wpc these monkeys had increased nAb yields compared to cohort 2 and 3. After challenge, mean nAbs of control monkeys rose continuously, reaching the maximum mean fold inhibition of 499.0 at 20 wpc. At the end of the observation period (28 wpc) cohort 1, 2 and 3 developed maximum mean fold inhibition of 733.3, 572.8 and 523.8, respectively.

### SIV env-specific IgG

**Figure 2:**
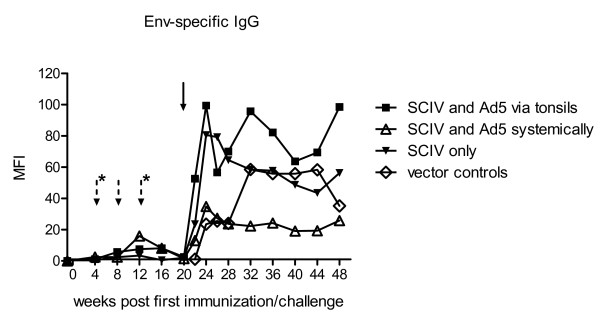
**IgG response to the viral env-proteins**. During vaccination, SIV-specific IgG antibodies targeting the envelope were determined in all vaccine groups and in the control group after challenge with SIVmac239. For this assay SIVmac251 infected HSC-F were incubated with heat-inactivated sera from vaccinated and infected animals. SIV-specific antibodies bound to infected T-cells were stained with a FITC-labelled anti-human IgG and determined by flow cytometry. Values are given as mean fluorescence intensities (MFI). Dotted arrows mark points in time of boosts and additional asterisks refer to boosts of group 1 only, whereas the black arrow indicates the point in time of challenge.

Hardly any SIV-specific IgG antibodies targeting the env were measured in vaccinated animals during the immunization period (Figure 2). The highest value measured was in monkeys of group 2 at 12 wpi, with a value of 16.0 MFI ± 15.6 (median: 10.9). Upon challenge, SIV-specific IgG antibody levels increased rapidly in monkeys of group 1 (maximum with 99.4 MFI ± 100.4 (median: 51.3)) and 3 (maximum with 80.7 MFI ± 29.8 (median: 85.1)), while those of group 2 were rather low but stable (ranging between 19.2 and 34.8 MFI) between 4 and 28 wpc. As expected, IgG antibody levels increased slowly in control animals. At 2 wpc, env-specific IgGs were significantly lower in controls when compared to immunized monkeys in all groups (p < 0.03); at subsequent points in time (4 and 8 wpc) controls showed minor differences with p-values being attenuated to borderline significance (p = 0.08 and p = 0.06, respectively) and the IgG-titres reached a maximum level of 58.5 MFI ± 39.2 (median: 50.3) at 12 wpc.

### Complement-mediated lysis

The contribution of C in reducing viral load was determined by lysis assays *in vitro*. Sera were collected before vaccination, directly before SIVmac239 challenge, 2 wpc and 28 wpc (Table 2). Before vaccination, complement-mediated lysis levels were below the detection limit of 10% in cohort 1, 2, and 3 (data not shown). Similarly, in control animals no lysis was measurable at the day of challenge. Simultaneously between 16% and 35% lysis was detected using sera of immunized monkeys. Notably, the lowest lysis results were measured in the orally immunized group 1 animals. Complement-mediated lysis levels were significantly higher in the immunized monkeys compared to controls by 20 wpi (all p < 0.05). Two weeks later, during peak viremia, sera of three orally immunized animals (#12127, #12128, #12131) still induced lysis levels lower than 30% (mean plasma RNA levels of group 1 = 3.2 × 10^4^log), while all except one monkey serum (#12142) of group 2 animals cleared between 40% and 96% of the input virus and cohort 2 exhibited mean plasma RNA levels of 2.5 × 10^4^log at that time. Similarly, sera harvested from animals of group 3 showed a clear increase in the lysis capacity and neutralized between 45% and 63% of the input virus. Samples from control monkeys induced mean lysis levels of 24.5% and had mean plasma RNA levels of 2.7 × 10^6^log ± 2.4 × 10^6^log (median: 1.6 × 10^6^log) at peak viremia. During the chronic phase, between 35% and 81% lysis (mean 58.5%) was measured in immunized monkeys; lysis levels in control monkeys ranged between 68% and 87% (mean 76.8%). Although the control animals exhibit a profound lysis capacity in the *in vitro *assay, the immunized animals had significantly lower mean plasma RNA levels (2.1 × 10^4^log ± 4.8 × 10^4^log (median: 3.6 × 10^3^log)) when compared to the levels in control monkeys (4.3 × 10^5^log ± 6.5 × 10^5^log (median: 8.0 × 10^4^log)) (p = 0.02). Differences between cohort 1 and 2 and cohort 2 and 3 were never statistically significant. Only at 2 wpc and 28 wpc were significantly higher lysis values observed in group 3 compared to group 1 (all p < 0.05). Thus, C-mediated lysis did not correlate with the control of virus replication *in vivo*.

**Table 2:** Induction of complement-mediated lysis

	monkey	%lysis day of challenge	%lysis 2 wpc	viral load 2 wpc^a^	%lysis 28 wpc	viral load 28 wpc^a^
group 1	12127	16	18	2.7 × 10^3^	49	1.8 × 10^3^
	
	12128	26	18	4.7 × 10^4^	41	6.6 × 10^3^
	
	12131	18	11	1.3 × 10^4^	51	3.1 × 10^3^
	
	12137	20	46	6.6 × 10^4^	53	20

group 2	12133	25	96	3.4 × 10^4^	35	1.9 × 10^3^
	
	12136	25	40	6.9 × 10^4^	66	1.1 × 10^4^
	
	12142	35	23	1.2 × 10^3^	58	2.1 × 10^4^
	
	12143	25	50	6.8 × 10^2^	68	> 10

group 3	12132	23	48	1.9 × 10^4^	81	1.8 × 10^5^
	
	12138	35	45	5.5 × 10^5^	63	4.1 × 10^3^
	
	12139	20	51	2.4 × 10^5^	66	2.4 × 10^4^
	
	12140	30	63	4.3 × 10^4^	71	7.7 × 10^2^

group 4	12129	< 10	12	9.0 × 10^5^	71	1.2 × 10^5^
	
	12130	< 10	18	6.7 × 10^6^	68	1.6 × 10^6^
	
	12134	< 10	28	2.1 × 10^6^	81	1.4 × 10^4^
	
	12141	< 10	40	1.0 × 10^6^	87	4.2 × 10^4^

^a^RNA copies/ml plasma

### Virus capture assay

**Figure 3:**
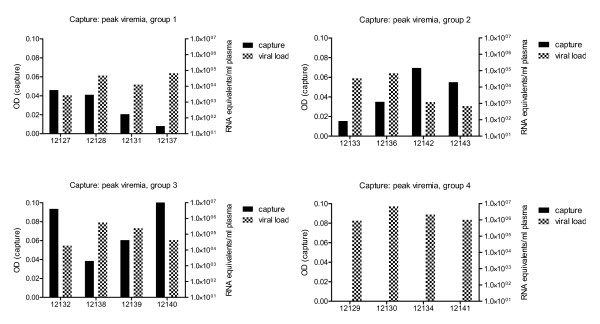
**Virus capture results at point in time of peak viremia**. Complement C3-deposition on viral particles is depicted on the left-y-axis and values are given as optical densities (OD). Plasma viral load levels are given on the right-y-axis and those exhibited a trend of an inverse correlation with C3 measured within the different cohorts at point in time of peak viremia.

For the virus capture assays, sera from immunized and SIV challenged animals were collected during peak viremia (2 wpc) and 28 wpc when the chronic infection was established. Interestingly, within the groups, most of the samples harvested during peak viremia exhibited a trend of an inverse correlation (Spearman correlation coefficient ranging between r_s _= -0.80 and r_s _= -0.60; p-values ranged between 0.2 and 0.4) when comparing C3-deposition on viral particles with plasma viral load (Figure 3). The immunized monkey (#12137) in group 1, which had the lowest C3-deposition at peak viremia, had plasma viral load levels of 6.6 × 10^4^log, while the animal with the strongest C3 signal (#12127) had a 1 log decreased viral load (2.7 × 10^3^log). Similarly, sera from the two animals (#12142, #12143) in group 2 with the lowest viral levels induced detectable C3-deposition. Within group 3, sera from monkey #12132 and #12140 showed more pronounced C3 levels on SIV and had plasma viral loads of 1.9 × 10^4^log and 4.3 × 10^4^log, respectively. The remaining four control monkeys had C3 levels below detection limit and a mean plasma viral load of 2.7 × 10^6^log at the point in time of peak viremia.

During chronic infection, the C3 opsonization was more pronounced when compared to the C3-deposition induced by sera collected during the peak viremia. However, the correlation between C3-deposition and viral load was no longer observable (data not shown).

## Discussion

In this study we analyzed the efficacy of humoral immune responses induced by different vaccination strategies either combining a SCIV [VSV-G] prime with an adenoviral boost or administering SCIV only (Table 1). The used SCIV [VSV-G] vaccine provides a safer immunization strategy when compared to live-attenuated vaccines, as no replication-competent particles are generated [15]. Adenoviral vectors have been used in the past, but were usually applied intramuscularly [22] and not via the tonsils. Although our approach did not induce sterilizing immunity, the vaccinated animals had a significantly reduced peak viremia after challenge with the highly pathogenic SIVmac239 when compared to the non-immunized but infected control animals. Peak viral load levels were reduced between 1 log in group 3 and 2 log in groups 1 and 2 (Figure 1A) [21]. Similar reductions in the viral titre were achieved by an iv prime-boost strategy using SCIV as a vaccine [23]. As many studies have emphasised that the long-term prognosis is significantly improved the lower the peak viral load levels are [24,25], the decrease of the viral load by oral administration of our vaccine may provide profound benefit.

**Figure 4:**
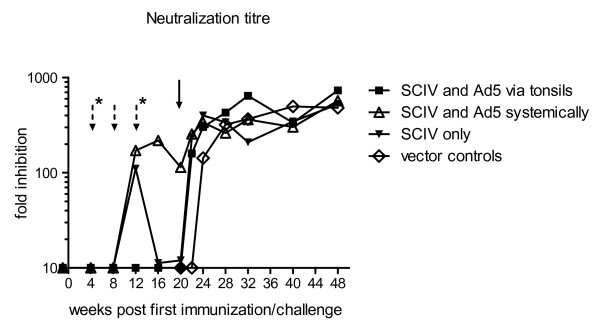
**NAb response determined by a yield reduction assay**. Before challenge (indicated by a black arrow) nAbs were measured in monkeys vaccinated with a heterologous prime-boost regimen (boosts are marked by dotted arrows, additional asterisks indicate boosts of group 1 only). After challenge nAbs were investigated for all four cohorts for the indicated period of time.

While vaccination via the tonsils induced no nAb responses before challenge, the prime-boost application of the vaccine iv and intramuscularly, respectively, resulted in detectable nAb-titres in the animals of group 2. Similar to the animals of group 1, the monkeys in group 3, which were primed by SCIV [VSV-G] and boosted with the MLV-pseudotyped SCIV, developed hardly any nAbs upon immunization (Figure 4). The peak viremia of group 3 was tenfold higher when compared to animals in group 1 or 2. Surprisingly, animals in group 1 or 2 controlled the viral replication to a comparable extent upon challenge with pathogenic SIV, although the vaccination in the tonsillar group induced no detectable nAb titres in the serum. However, the Ab levels in this group increased rapidly after challenge and reached a constant high titre already 2 wpc. Additionally, the application of the vaccine via the tonsils may induce IgA or cytotoxic T-lymphocyte response at the mucosal site, which may contribute to the reduction of the viral titre upon tonsillar challenge with SIVmac239. Unfortunately, we were not able to measure IgA responses of these vaccinated animals. The presence of nAbs before challenge and/or their fast induction after challenge may contribute to the decrease of the virus in the plasma. This would be in line with reports indicating that only high concentrations of nAbs reduce the peak viremia [26,27].

Along with the nAb titres, the levels of the total env-specific IgG were weak but mainly detectable in the systemically immunized animals of group 2 already 12 wpi. The detection of the Abs by FACS analysis using SIV-infected cells allows the detection of native, *in vivo *accessible epitopes only and may be less sensitive compared to ELISA detection systems. Stahl-Hennig et al. [21] used a gp130 ELISA with proteins expressed in E. coli for this animal study. However, these proteins do not reflect the *in vivo *conformation of the env-protein complex and may thus account for overestimated IgG titres and explain the controversial findings reported previously [21]. It is possible that neutralizing antibodies are not detected by FACS, but will be recognized in ELISA assays. One example is the monoclonal antibody 2F5 [28] which binds to the membrane proximal external region of gp41 during the fusion process but not in the native state. After infection with SIVmac239, the overall IgG response was dramatically boosted in all animals and ran parallel to the induction of nAbs. Interestingly, group 1 and 2 which both controlled the virus similarly well exhibited marked differences in the amount of total env-specific IgG. Due to the limited number of animals available for this study, these differences in the IgG titres reached significance only at week 28.

A neonatal macaque study showed that passively transferred non-nAbs did not protect the animals against oral challenge with SIVmac251 indicating that ADCC is not a main mechanism in reducing infection [29].

This is in contrast to recently reported findings which indicate that ADCC or the interaction of FcR with the Fc-region of the Abs may contribute to the elimination of retroviral infections [8,30].

Furthermore, the data presented in the present study suggests that C activation is part of the humoral immune response. As shown by a virus capture assay, sera of the animals collected at 2 wpc induced C3-deposition on the viral surface. Although based on only four animals per group, a trend to an inverse correlation of C3-deposition on viral particles and viral load during peak viremia was observed at least within the individual groups of vaccinated monkeys (Figure 3). During the chronic phase of infection, sera of all vaccinated macaques induced C3 activation and opsonisation on SIV, independent of the viral load. C-mediated defence mechanisms have been discussed controversially in the literature. Opsonized virus particles may interact with C-receptor expressing cells, such as B-cells or dendritic cells [31-34], followed by an efficient transmission of opsonized HIV to autologous primary T-cells. At least *in vitro*, the infection is significantly enhanced by this mechanism. However, preliminary data indicate that in *in vitro *interaction assays the C-mediated increase of SIV infection is not observable in the monkey system using primary isolated macaque B- and T-cells and opsonised SIV (unpublished observation). A further mechanism of C to reduce infectivity of C-receptor-negative T-cells is the masking of viral epitopes due to the deposition of C3-fragments on the viral envelope [35,36]. This neutralization mechanism has also been described for other viruses [37] and is an attractive hypothesis to explain, at least in part, the reduced viral loads observed during peak viremia.

A further result of C activation is the induction of the terminal C pathway, resulting in the destruction of pathogens. The *in vitro *lysis assays reduced the viral titres by a mean of 24.8% (range between 16 and 30%) when sera of immunized monkeys were tested before challenge (Table 2). Two weeks later, during peak viremia, mean lysis was 38.0% (ranging between 11 and 96%) tested in control and vaccinated monkeys. Lysis values increased further during chronic infection up to mean levels of 63.1% (range between 35 and 87%). Although C-induced lysis may contribute to the control of SIV replication, C-mediated destruction of the virus did not correlate with the control of the infection *in vivo*. Some animals had low peak viremia (#12127, #12142) but exhibited a poor induction of C-mediated lysis when compared to sera from other monkeys with extremely high lysis activities (#12133, #12140) but ten times higher viral loads. In line with earlier studies [11,12,38], no correlations between nAbs and C-mediated lysis was observed during the chronic phase of infection. Thus, Ab-mediated neutralization and C-induced lysis of retroviruses appear to represent two independent parameters which are not necessarily linked [38]. This does not exclude the possibility that lysis may play an important role during early phases of infection before or early after seroconversion [13].

Beside Abs, effective SIV-specific T-cell responses are important for controlling viremia [39]. Recently published INF-γ ELISPOT data from the present vaccination trial revealed increased cellular immune responses in cohort 2 compared to group 1 [21]. As both groups controlled the viral loads at comparable levels, it is presently unclear to which extent the cytotoxic T-lymphocyte response is the main contributor for the reduced peak viremia and viral load reduction in the chronic phase of infection.

## Conclusion

With this rhesus macaque study it was demonstrated that priming with SCIV [VSV-G] and boosting with both Ad5-SIV vectors or SCIV [MLV] mount humoral immune responses comparable to that of live-attenuated immunodeficiency virus vaccines [40,41], which may contribute to the significant reduction in viral load observed in animals of group 1 and 2 after challenge. This encourages tonsillar/mucosal immunization strategies which may simplify vaccine application in the future. Thus, more efforts in research further investigating this mucosal delivery route are warranted.

## Materials and methods

### Animals

Young adult rhesus monkeys (Macaca mulatta) were imported from China through R.C. Hartelust BV, Tilburg, the Netherlands. Monkeys of both sexes were antibody negative for simian T-lymphotropic virus type 1, simian D-type retrovirus and SIV. Viral application, physical examinations and bleeding were done under ketamine anaesthesia. The nonhuman primate study was performed at the German Primate Centre according to paragraph 8 of the German Animal Protection law which complies with EC Directive 86/609, with project licence 509.42502/08-04.03 issued by the District Government Braunschweig, Lower Saxony.

### Vaccination strategies, challenge and specimen collection

The study was conducted on 16 monkeys (Table 1). In group 1, four macaques were immunized with SCIV [VSV-G] [42] via tonsillar spray application at 0 and 4 wpi, as described recently [16,43], and boosted by the same route with Ad5-SIV expressing gag-pol or env-rev at 8 and 12 wpi. Group 2 consisted of four monkeys which were immunized iv with SCIV [VSV-G] and boosted intramuscularly with Ad5-SIV 8 wpi. In group 3, four monkeys were primed with SCIV [VSV-G] iv and boosted with SCIV [MLV] iv at 8 wpi. SCIV [MLV] were prepared as described for SCIV [VSV-G] by just replacing the VSV-G expression plasmid by pHIT456 [44], an expression plasmid for amphotropic MLV env. Group 4 monkeys served as controls, two (#12129 and #12130) of which were immunized with an adenoviral vector containing a green fluorescent protein gene (Ad5-GFP) [45] via the tonsils at 8 and 12 weeks after the initiation of the experiment. The other two controls (#12134 and #12141) were immunized with Ad5-GFP intramuscularly at week 8. All macaques were challenged with approximately 2000 TCID_50 _of SIVmac239 [46,47] via the tonsils 20 wpi. Sera from vaccinated and control animals were collected periodically as indicated in the figures. The heat-inactivated (hi; 56°C, 30 min) serum samples of the monkeys were used to analyze for Ab responses. As a source of complement, a pool of normal monkey serum (NMS) from untreated donors was used.

### Determination of viral loads

Viral RNA in plasma was determined by quantitative real-time PCR as previously reported [17]. In order to quantify plasma viral load, standard RNA templates were generated from the p239Sp5' plasmid (kindly provided by R. M. Ruprecht, Dana-Farber Cancer Institute, Boston, USA; [48]) with a detection limit of 10 viral particles per ml of plasma.

Cell-associated virus loads were determined by a limiting dilution co-culture assay with mononuclear cells from blood as described previously [16,40,41].

### SIV p27 antigen assay

SIVmac251 replication was determined by ELISA against the p27 core protein as described recently [41].

### SIV neutralization assays

Levels of nAbs against SIVmac251 in the sera of immunized and infected macaques were measured using a yield reduction assay [42]. Briefly, sera diluted 1:50 were incubated with serial dilutions of SIVmac251 (25 μl serum, 25 μl virus, six replicates per dilution) in U96 microtitre plates (1 hour at 37°C). Then 150 μl of a C8166 cell suspension (2000 cells) was added. The cultures were lysed after a 7 day incubation at 37°C and virus replication in individual wells was measured by a sensitive gag-based antigen capture ELISA. Wells, giving OD values above threshold (mean of uninfected wells + 5× standard deviations), were scored positive, and the TCID_50 _for the virus in the presence of each serum was calculated. The yield reduction for each sample was then calculated as the virus titre in the absence of serum divided by the titre in the presence of serum.

### Measurement of SIV-specific IgG

Flow cytometry was used to evaluate SIV-specific IgG responses. HSC-Fcells (provided by the EU-program EVA/MRC (QLKZ-CT-1999-00609)) [49] were infected with SIVmac251. After washing, cells (5 × 10^5^/analysis) were incubated on ice with hi-sera from vaccinated and infected animals (1:50, 30 minutes, two replicates per sample performed in duplicate). SIV-specific antibodies bound to infected cells were stained with a FITC-labelled anti-human IgG (Dako F0202, Glostrup, Denmark). As a negative control, hi-NMS of healthy untreated donors was used. Samples were analysed by flow cytometry using Cell Quest software (Becton Dickinson, Franklin Lakes, New Jersey, USA). Data given in the figures represent mean-fluorescence intensities (MFIs).

### Lysis assay

Hi-sera of immunized rhesus macaques (1:50, two replicates per sample performed in double) were incubated with SIVmac251 (40 ng/ml p27, TCID_50 _= 1.5 × 10^5^log) for 30 minutes at 4°C. Subsequently NMS was added (1:10, 30 minutes at 37°C) as a source of C. The viral RNA accessible due to the formation of the membrane attack complex was digested by the addition of RNAse. As a negative control, NMS was replaced by hi-NMS or RPMI1640 medium without any supplements (background lysis). As a control for 100% lysis, SIV was incubated with 1% of Igepal (Sigma, Vienna, Austria). Samples were centrifuged (13.000 rpm, 90 minutes at 4°C) and RNA from non-lysed pelleted SIV was extracted using QIAamp^® ^Viral RNA kit (Qiagen, Valencia, California, USA) according to the manufacturer's instructions. Remaining intact virus was quantified by real-time reverse transcriptase PCR (iCycler, BioRad, Hercules, CaliforniaA, USA) using the iScript™ One-StepRT-PCR Kit (Bio-Rad, Hercules, California, USA) as previously described [17]. As the efficacy of the PCR is close to 100%, a decrease of 3 threshold cycles (Ct) in the real-time PCR corresponds to reduction of 1 log in the viral titre. Thus, a decrease of 1Ct-value corresponds to approximately 33% lysis and was calculated as follows:



### In vitro opsonisation and virus capture assay

Hi-monkey samples (1:50, two replicates per sample performed in double) from the vaccinated, and infected animals were incubated with SIVmac251 (160 ng/ml p27, TCID_50 _= 5.9 × 10^5^log) for 30 minutes at 4°C in order to allow for the binding of the induced env-specific IgGs. Subsequently, NMS was added in a 1:10 dilution as a source of C. Hi-NMS was used as control. Samples were further incubated for 30 minutes at 37°C. To remove unbound antibodies and remaining C proteins, the virus was pelleted and re-dissolved in RPMI1640 medium. The opsonisation of the virus with C3 fragments was determined by a virus capture assay as described previously [50]. Depending on the amount of C3 deposited on the viral surface, opsonised virus was retained in the ELISA plate. Virus was lysed by RPMI/1%Igepal and quantified by a p27-ELISA.

### Statistical analysis

Continuous data are presented as means ± standard deviations, with medians in parenthesis. Kolmogorov-Smirnov-tests were conducted in order to test for Gaussian distribution of plasma and cell-associated viral load, nAbs, SIV-specific IgG titres, lysis, as well as capture parameters. Since the above variables showed significant deviation from normality at an Alpha-Level of 0.05, non-parametric tests were used throughout the analyses. We used the Kruskal-Wallis-H-Test to assess overall differences between control monkeys and immunized groups, with post-hoc Mann-Whitney-U-Tests to compare pair-wise differences between groups. Non-parametric Spearman correlation was used to investigate associations of lysis parameters. Two-sided p-values < 0.05 were considered statistically significant. All statistical analyses were conducted using SPSS 15.0 (SPSS Inc., Chicago, Illinois, USA).

## Abbreviations

Abs: antibodies; ADCC: antibody dependent cellular cytotoxicity; C: complement; env: envelope; hi: heat-inactivated; MFI: mean fluorescence intensities; MLV: murine leukemia virus; nAbs: neutralizing antibodies; NMS: normal monkey serum; SHIV: SIV/HIV hybridvirus; SIV: simian immunodeficiency virus; TCID_50_: median tissue culture 50% infectious dose; VSV-G: G protein of vesicular stomatits virus; wpc: weeks post challenge; wpi: weeks post immunization;

## Competing interests

The authors declare that they have no competing interests.

## Authors' contributions

BF, BR, AH and SN carried out the experiments and analysed the data. DW determined plasma viral load levels and performed the statistical analysis together with AS. CSH took care of the rhesus monkeys, took blood samples from the animals regularly, measured cell-associated viral load levels and corrected the manuscript. SK and KÜ designed the vaccines and corrected the manuscript. PR and HS conceived of the study, and participated in its design and coordination. HS and BF wrote the manuscript. All authors read and approved the final manuscript.
